# *N*-methylsansalvamide elicits antitumor effects in colon cancer cells *in vitro* and *in vivo* by regulating proliferation, apoptosis, and metastatic capacity

**DOI:** 10.3389/fphar.2023.1146966

**Published:** 2023-03-16

**Authors:** Juhee Park, Sung-Kwon Moon, Chan Lee

**Affiliations:** ^1^ Food Analysis Research Center, Food Industry Research Division, Korea Food Research Institute, Wanju, Republic of Korea; ^2^ Department of Food Science and Biotechnology, Chung-Ang University, Anseong, Republic of Korea; ^3^ Department of Food and Nutrition, Chung-Ang University, Anseong, Republic of Korea

**Keywords:** N-methylsansalvamide, colon cancer, G0/G1 cell cycle, apoptosis, migration, invasion, *in vitro* metabolism, xenograft mice

## Abstract

*N*-methylsansalvamide (MSSV), a cyclic pentadepsipeptide, was obtained from a strain of *Fusarium solani* f. radicicola. The current study investigated the anti-colorectal cancer effect of MSSV. MSSV exhibited the inhibition of the proliferation in HCT116 cells *via* induction of G0/G1 cell cycle arrest by downregulating CDK 2, CDK6, cyclin D, and cyclin E, and upregulating p21WAF1 and p27KIP1. Decreased phosphorylation of AKT was observed in MSSV-treated cells. Moreover, MSSV treatment induced caspase-mediated apoptosis through elevating the level of cleaved caspase 3, cleaved PARP, cleaved caspase 9, and pro-apoptotic Bax. MSSV revealed the declined MMP-9 level mediated by reduction in the binding activity of AP-1, Sp-1, and NF-κB motifs, which led to the migration and invasion of HCT116 cells. *In vitro* metabolism with rat liver S9 fractions was performed to examine the effect of MSSV metabolites. The metabolic process enhanced the inhibitory effect of MSSV on the HCT116 cell proliferation *via* decline of cyclin D1 expression and AKT phosphorylation. Finally, oral administration of MSSV inhibited the tumor growth of HCT116 xenograft mice. These results suggest that MSSV is a potential anti-tumor agent in colorectal cancer treatment.

## 1 Introduction

Globally, 1.8 million new cases of colorectal cancer (CRC) are diagnosed annually (Keum and Giovannucci, 2019). CRC is the second and third most common cancer in women and men, respectively (Tariq and Ghias, 2016). Owing to the often late diagnosis at advanced clinical stages, about 900,000 individuals die from CRC (Keum and Giovannucci, 2019). The incidence rates are higher in developed countries than in developing countries. Nevertheless, the incidence of CRC is rapidly rising in developing countries due to economic development and changes in diet and lifestyle, and its mortality is higher in these countries (Favoriti et al., 2016; Tariq and Ghias, 2016; Keum and Giovannucci, 2019).1–3. The pathogenesis of CRC is influenced by multiple factors such as diet, lifestyle, sex, age, etc (IARC, 2019; Keum and Giovannucci, 2019). Surgical resection or radiotherapy combined with chemotherapy are the standard of care for CRC. These treatments enhance survival rates and decrease the quality of life of patients with CRC (Liu et al., 2014). In addition, the increasing side effects of drug treatment cause frequent changes in the lines of treatment leading to multidrug resistance (Tyagi et al., 2015; Chiangjong et al., 2020). Anti-cancer peptides (ACPs) could be used as a new therapeutic strategy against cancer cells (Chiangjong et al., 2020).

**FIGURE 1:**
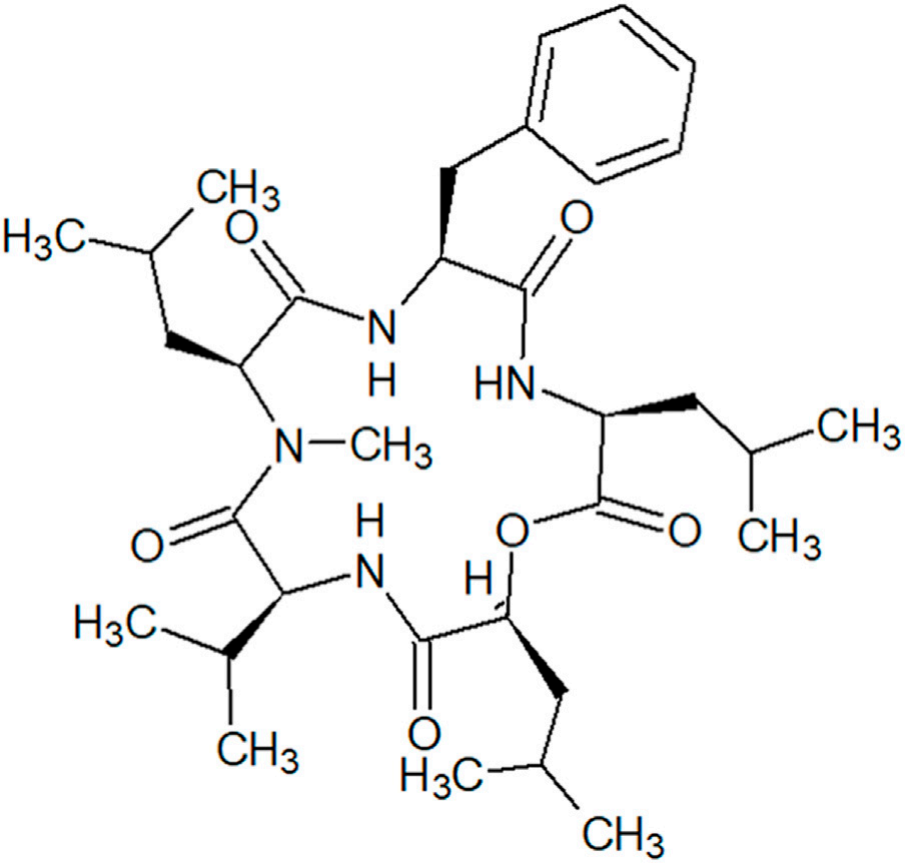
Structure of *N*-methylsansalvamide.

ACPs are a better choice of therapeutics compared to antibodies and small molecules because of their small size, ease of synthesis and modification, good biocompatibility, and tumor penetrating ability (Thundimadathil, 2012). Diverse cyclic peptides possess anti-tumor properties in CRC cell lines due to their different structures and various mechanisms (Wesson and Hamann, 1996; Simmons et al., 2009). In our study, *N*-methylsansalvamide, a cyclic pentadepsipeptide and a *N*-methylated analogue of Sansalvamide A (San A), was observed as a metabolite of *Fusarium* solani isolated from a potato grown in South Korea. MSSV was first identified from a *Fusarium* species (strain CNL-619), which was isolated from the Caribbean green algae *Avrainvillea* sp. Collected from a mangrove in the United States Virgin Islands in 1996 (Cueto et al., 2000). This cyclic pentadepsipeptide consisted of Val, Leu, Phe, *N*-methyl leucine (*N*-Me-Leu), and leucic acid (*O*-Leu) (Figure 1) with moderate cytotoxicity in the National Cancer Institute’s human tumor cell line screen. Moreover, the extracts obtained from the mycelium and broth of *Fusarium* sp., strain CNL-619, exhibited *in vitro* cytotoxicity against human CRC HCT116 cells (Cueto et al., 2000). However, its cytotoxic mechanisms remain unknown.

In a previous study, several San A analogues with *N*-methylation and para-bromination showed potent anti-cancer activity for growth inhibition of human pancreatic cancer cells. Among these, a Sansalvamide analogue inhibited cell proliferation with G0/G1 phase arrest and apoptosis (Ujiki et al., 2006). Our previous study reported the anti-tumor action of this analogue against bladder cancer and anti-angiogenic responses both *in vitro* and *in vivo* (Song et al., 2021). Extracts containing MSSV have already been shown to be effective against CRC cell lines (Cueto et al., 2000); however, its relevant molecular mechanism has never been investigated. Thus, we herein report the detailed mechanism of action of MSSV, which is involved in the inhibition of HCT116 cell responses using *in vitro* and *in vivo* systems.

## 2 Materials and methods

### 2.1 Chemicals and antibodies

In current study, MSSV was isolated from *Fusarium* sp. FB900, which was grown in *Fusarium* defined agar media at 25°C. It was extracted from a medium containing fungal strains with chloroform and evaporated using a rotary evaporator. The dried extract was dissolved with methanol and the crude extract was fractionated using vacuum liquid chromatography (VLC). VLC was carried out with gradient elution of acetone and n-hexane. Specific fractions containing MSSV were evaporated using a rotary evaporator. Purified MSSV was then used in this study.

MTT [3-(4,5-dimethylathiazol-2-yl)-2,5-diphenyltetrazolium bromide] and dimethyl sulfoxide (DMSO) were purchased from Sigma-Aldrich (St. Louis, MO, United States). We obtained 0.25% Trypsin containing 2.21 mM EDTA from Corning (Manassas, VA, United States).

Monoclonal antibodies against Cyclin D1, Cyclin E, CDK2, CDK6, Bax, Caspase 3, Caspase 9, and MMP-9 were purchased from Santa Cruz Biotechnology (Santa Cruz, CA, United States). AKT, p-AKT, p21Waf1, p27KIP1, PARP, β-actin were products of Cell signaling technology (Danvers, MA, United States).

### 2.2 Cell culture

The human CRC cell line HCT116 was purchased from the American Type Culture Collection (ATCC) (Manassas, VA, United States). HCT116 was cultured in Dulbecco’s modification of eagle’s medium (DMEM, Corning, Manassas, VA, United States) and supplemented with 10% heat-inactivated fetal bovine serum (FBS, Corning, Manassas, VA, United States). Cells were maintained at 37°C in a humidified atmosphere containing 5% CO_2_.

### 2.3 The MTT assay

The MTT assay was conducted to measure cellular proliferation with MTT [3-(4,5-dimethylathiazol-2-yl)-2,5-diphenyltetrazolium bromide]. HCT116 cells were seeded on a 96-well cell culture plate. After culture for 24 h, cells were treated with MSSV (0, 1, 15, 20, and 25 μM) and the plate was incubated at 37°C for 24 h. The culture media was replaced with fresh media containing MTT solution for 2 h. The media was removed from the culture plate and purple formazan was dissolved using 200 μL of DMSO in each well by gentle mixing. The absorbance of colored media was determined at a wavelength of 570 nm using a SpectraMax 190 microplate reader (Molecular devices, United States).

Furthermore, a cell counting assay was performed using the trypan blue staining method. HCT116 cells plated on a 6-well cell culture plate were incubated for 24 h and were treated with different concentrations of MSSV (0, 10, 12.5, 15, 17.5, and 20 μM) for 24 h at 37°C. The cells were detached from the plate by treatment with 0.25% trypsin containing 2.21 mM EDTA. The detached cells were mixed with the same amount of trypan blue solution (Sigma Aldrich, St. Louis, MO, United States) by gentle pipetting. The mixture was loaded into each chamber of a haemacytometer and counted.

### 2.4 The colony formation assay

HCT116 cells (3×10^3^ cells/well) were seeded on a 6-well culture plate and incubated for 24 h. The cells were incubated for 10 days with the media containing MSSV (5, 7.5, 10, 12.5, and 15 μM) at 37°C in an atmosphere containing 5% CO_2_. After 10 days, the media was removed and cells were rinsed with phosphate buffered saline (PBS) (AMRESCO, OH, United States) twice and fixed with methanol for 15 min. The fixed cells were stained with 0.5% crystal violet solution (ACROS organics, New Jersey, United States) for 10 min and rinsed with PBS. The stained colonies were photographed using a microscope (Olympus CK2) equipped with ProgRes^®^ SpeedXTcore 3 (Olympus optical Co., Ltd., Japan).

### 2.5 The wound-healing migration assay

Cell migration abilities on 2D surfaces were evaluated by a wound-healing migration assay. Cells were plated on a 6-well cell culture plate, and incubated culture media to 90% confluence. The monolayer cells were scratched with pipette tip triplicates in each well and then washed with PBS carefully. The plate was incubated with media in the presence or absence of MSSV (0, 5, 7.5, and 10 μM) for 24 h. The cells were moved to close the wound during incubation. Random fields on each well were photographed at 0 h and 24 h after scratching. The wound area was analyzed using the ImageJ software (NIH).

### 2.6 The boyden chamber invasion assay

Boyden chamber invasion assay, also called transwell invasion assay, was performed to assess the invasive ability on 3D matrix of MSSV on HCT116 cells (Kramer et al., 2013). The chamber with polycarbonate membranes with 8 μm pore sizes were placed on 24-well cell culture plate, and its bottom layer was coated with 10 μL of gelatin solution to form a thin layer of extracellular matrix (ECM). The HCT116 cells (7×10^4^ cells/well) were plated into the upper chamber and treated with different concentrations of MSSV diluted with serum-free media in the upper chamber. In the lower chamber FBS was used at 10% concentration as a chemo-attractant. After 24 h incubation, the culture media was removed from the chamber and well. The cells were rinsed with PBS and fixed with 4% paraformaldehyde (PFA) for 15 min. After fixing, the cells were washed with PBS twice and stained with 0.5% crystal violet solutions for 10 min. The cells were rinsed with PBS and then the non-invaded cells on the inner layer of the upper chamber were carefully removed with a cotton swab. The invaded cells which stayed on the upper side of upper chamber were detected using a microscope. Three random fields were photographed, and the density of the stained cells were estimated using the ImageJ software.

### 2.7 Gelatin zymography

The HCT116 cells (3.2×10^5^ cells/plate) were plated on a 60 mm diameter cell culture dish. The cells were incubated with media in the presence or absence of MSSV (0, 5, 7.5, and 10 μM) for 24 h. The cellular supernatant was prepared from each treatment concentration, which were loaded on a polyacrylamide gel containing 0.25% gelatin. After electrophoresis, the gel was incubated with incubation buffer (1M Tris-HCl adjusted to pH 7.5, 1M CaCl_2_, and distilled water) at 37 °C overnight. MMP-9 band density was quantified using the Gel Doc^TM^XR + Imaging system (Bio-rad, Hercules, CA, United States).

### 2.8 Cell cycle distribution analysis

Cells were treated with various concentrations of MSSV (0, 10, 15, and 20 μM) for 24 h. The cells (1×10^6^ cells) were fixed with 70% ethanol and rinsed with PBS twice. The washed cells were treated with the Muse^®^ cell cycle kit (Luminex, Austin, TX, United States) according to the manufacturer’s instructions. The cell cycle distribution affected by treatment of MSSV was analyzed using a Muse^®^ Cell Analyzer (EMD Millipore Corporation, Hayward, CA, United States).

### 2.9 Cell apoptosis by flow cytometry

HCT116 cells (3.2 × 10^4^ cells/dish) were seeded on a 60 mm diameter cell culture dish. The cells were incubated in the presence or absence of MSSV (0 and 40 μM) for 24 h. A BD FACSAria^TM^II Cell Sorter (BD Biosciences, San Jose, CA, United States) was set with parameters including events to record (5,000 evt), FSC (148 V), FITC (330 V), flow rate (1 mL/min), PE (320 V), and SSC (330 V). The cells were stained with FITC Annexin V and propidium iodide (PI) staining solutions for 15 min in the dark, in accordance with the instructions of the FITC Annexin V Apoptosis Detection Kit I (BD Biosciences, San Diego, CA, United States. Cellular apoptosis was determined using the DIVA software.

### 2.10 Immunoblotting

Cells were treated with MSSV for 24 h and lysed using Pierce^®^ RIPA buffer (Thermo scientific, Rockford, IL, United States). The lysates were incubated on ice for 15 min and then centrifuged at 15,000×*g* for 15 min at 4°C. The quantitation of supernatant was estimated using Bicinchoninic acid and copper (II) sulfate solution (Sigma-Aldrich, St. Louis, MO, United States). The crude cell extract was resolved by sodium dodecyl sulfate-polyacrylamide gel electrophoresis (SDS-PAGE), which was transferred onto nitrocellulose membranes (Hybond, GE Healthcare Bio-Sciences, Marlborough, MA, United States). After transference and blocking, membranes were incubated with the following primary antibodies; p21Waf1, p27KIP1, β-actin, AKT, p-AKT, ERK, p-ERK, eNOS, and p-eNOS, Cyclin D1, Cyclin E, CDK2, CDK6, Bax, caspase 3, caspase 9, cleaved PARP, and MMP-9. Proteins were visualized on medical X-ray film blue (Alfa Healthcare NV, Mortsel, Belgium) with Amersham™ ECL Select™ Western blotting detection Reagent (GE Healthcare, Buckinghamshire, United Kingdom) and/or Clarity™ Western ECL substrate (Bio-rad, Hercules, CA, United States). Quantifications of protein were conducted using the ImageJ software.

### 2.11 The electrophoretic mobility shift assay (EMSA)

HCT116 cells were incubated at the indicated concentrations of MSSV. After 24 h, cells were washed, centrifuged (2,264 × *g*, 4°C, 5 min), collected, and cell lysates were obtained by the EMSA lysis buffer A [10 mM KCl, 10 mM HEPES (pH 7.9), 1 mM DTT, 0.1 mM EGTA, 0.5 mM PMSF, and 0.1 mM EDTA]. The cell lysates were added to NP-40 (0.5%, 20 μL), and then followed by centrifugation. The supernatant was removed and the pellets were incubated with the EMSA lysis buffer B [400 mM NaCl, 20 mM HEPES (pH 7.9), 1 mM PMSF, 1 mM EGTA, 1 mM DTT, and 1 mM EDTA]. After centrifugation, the nuclear extracts were obtained and quantified using the commercial BCA Protein Assay Reagent Kit (Thermo Fisher Scientific). The sequences of the oligonucleotides applied were as follows: Sp-1, ATT​CGA​TCG​GGG​CGG​GGC​GAG​C; NF-κB, AGT​TGA​GGG​GAC​TTT​CCC​AGG​C; and AP-1, CGT​TGA​TGA​GTC​AGC​CGG​AA (Macrogen Corp., Seoul, Korea). An EMSA binding buffer [(25 mM HEPES buffer (pH 7.9), 0.5 mM EDTA, 0.5 mM DTT, 50 mM NaCl, and 2.5% glycerol)] containing Klenow end-labeled (32P ATP) 30-mer oligonucleotide and 2 μg poly dI/dC was incubated with the nuclear extracts for 20 min at 25°C. The probe-binding nuclear proteins were separated by 6% PAGE at 4°C. The gels were exposed to an X-ray film, and the visualized band intensities were quantified using the ImageJ program (NIH).

### 2.12 *In vitro* metabolism of MSSV with S9 fraction on HCT116 cells

*In vitro* metabolism was carried out with S9 fractions to examine the effect of compound metabolites on cytotoxicity and protein expression. Sprague-Dawley rat pooled liver S9 was a product of Corning (Tewksbury, MA, United States). NADPH tetrasodium salt was obtained from Roche (Darmstadt, Germany). Glucose-6-phosphate (G-6-P), glucose 6-phosphate dehydrogenase (G-6-PD), L-glutathione reduced (GSH), magnesium chloride hexahydrate (MgCl_2_), adenosine 3′-phosphate 5′-phosphosulfate lithium salt hydrate (PAPS), and uridine 5′-diphosphoglucuronic acid trisodium salt (UDPGA) were purchased from Sigma-Aldrich (St. Louis, MO, United States). All chemical mentioned above were dissolved and diluted in water at stock concentrations.

HCT116 cells were plated on a 24-well culture plate. After incubation for 24 h, the cells were treated in the presence or absence of MSSV for 24 h, and were classified into three groups as follows: parent, phase I metabolism, and phase I + II metabolism. The cells of the Parent group were treated with various concentrations of MSSV (0, 10, 12.5, 15, 17.5, and 20 μM). The Phase I metabolism group was treated with MSSV metabolites reacted with 0.01 mg/mL rat liver S9 fraction, 3 × 10^−3^ M G-6-P, 3 × 10^−1^ units/mL G-6-PD, 5 × 10^−3^ M MgCl_2_, and 2 × 10^−4^ M NADPH tetra sodium salt. In the Phase I + II metabolism group, cells were incubated with MSSV metabolites reacted with the same substances treated to the Phase I metabolism group, and 2 × 10^−3^ M GSH, 2 × 10^−6^ M PAPS, and 5 × 10^−4^ M UDPGA were supplemented into the Phase I + II metabolism group. The cells were detached from each well and then mixed with equal volumes of trypan blue solution (Sigma-Aldrich, St. Louis, MO, United States) by gentle pipetting. The number of cells were counted to determine the effect of *in vitro* metabolism in HCT116 cells. The cells used in estimation of protein expression were prepared with both concentrations of MSSV (10 and 20 μM). Protein quantification was carried out in the same way described in *2.10. Immunoblotting*.

### 2.13 Animal experiment and histological examination

BALB/c nude mice (5 weeks old male) were purchased from DBL Co., Ltd. (Eumseong, Korea) and used for the xenograft mouse model. The Animal Care and Use Committee of Chung-Ang University followed (EBOA-2017-12) and approved the entire experimental protocol (EBO17104). HCT116 cells (4 × 10^6^ cells/mouse) were inoculated subcutaneously into the right dorsal skin of each nude mice. Mice were subjected to oral injections of various concentrations of MSSV (5 and 15 mg/kg) in 0.1% DMSO solution daily for 21 days. A positive drug (5 mg/kg cisplatin) was also administered to the mice to compare its efficacy with that of MSSV. To compare efficacy of MSSV, a positive drug (5 mg/kg cisplatin) was also injected *via* intraperitoneal administration. Individual body weights were measured daily. After 20 days, the mice were anaesthetized and the tumor tissues were isolated and weighed. For histological examination, tumor tissues were fixed, dehydrated, embedded in paraffin, and then sectioned (5-μm thickness) using a rotary microtome. The sections were stained with the Ki-67 antibody (eBioscience™, Invitrogen, United States), mounted, and brown staining located in nucleus was observed under a microscope for histopathological evaluation.

### 2.14 Statistical analysis

All results are presented as the mean ± standard deviation (S.D.) from three independent experiments. One-way analysis of variance (ANOVA) was performed to assess the statistical significance of the mean difference between more than two different groups, followed by the Dunnett’s test. A *p*-value < 0.05 was considered statistically significant.

## 3 Results

### 3.1 MSSV inhibits the cell proliferation of HCT116 cells

**FIGURE 2:**
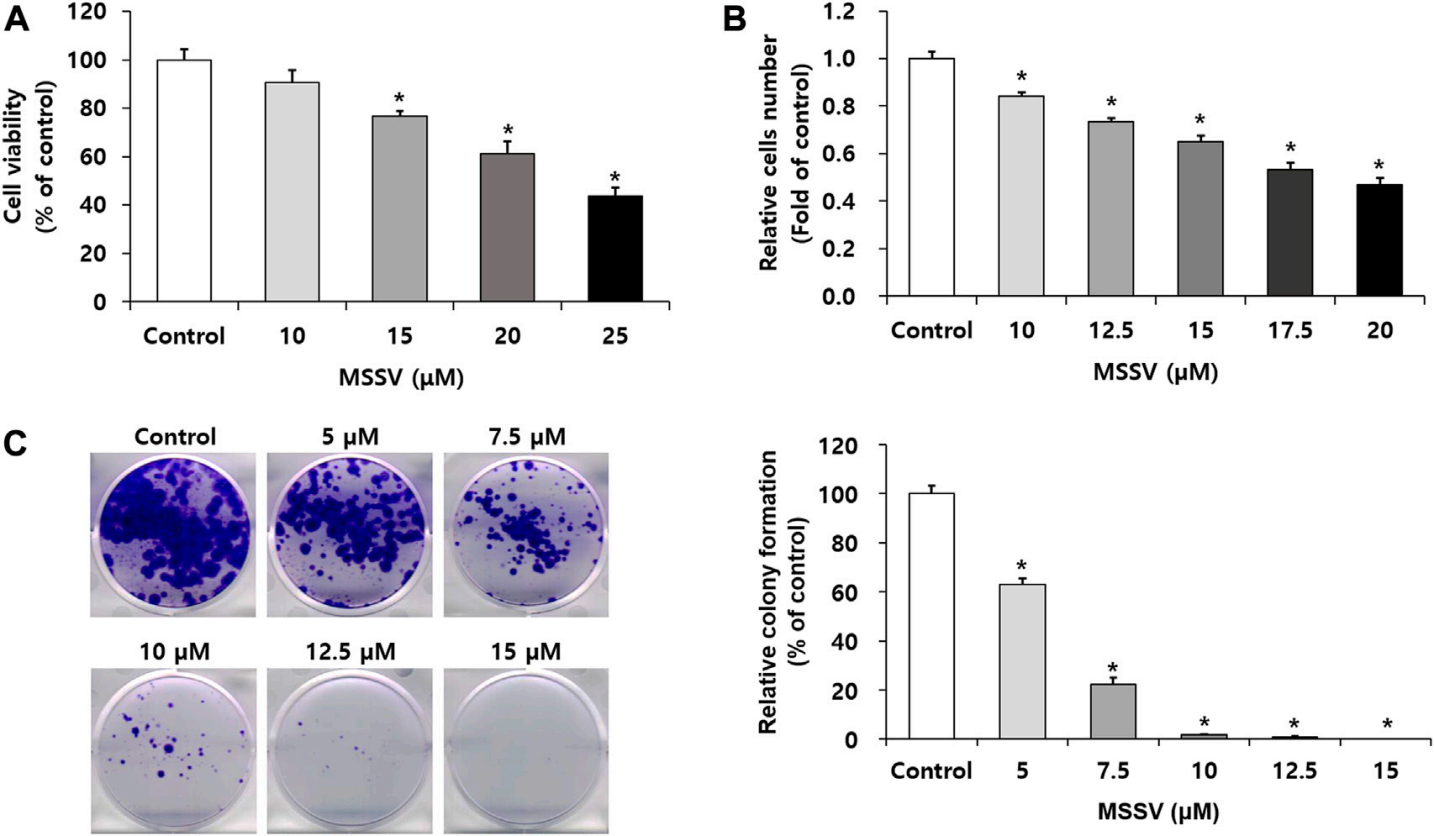
MSSV inhibits the proliferative capacity against CRC cells **(A)** HCT116 cells were incubated in the presence or absence of MSSV for 24 h. Cell viability was estimated by an MTT assay. **(B)** A cell counting assay was performed to determine the anti-proliferative activity. Trypan blue staining method was applied for the estimation of cell proliferation. **(C)** The inhibitory effects of MSSV on the colony formation of HCT116 cell. HCT116 cells were incubated in the presence or absence of MSSV on a 6-well plate for 12 days, followed by colony formation. Data are presented as mean ± SD and the * (asterisk) indicates a statistically significant difference. A *p*-value of <0.05 was considered statistically significant.

We investigated the anti-proliferative effect of MSSV on HCT116 CRC cells by MTT, cell counting, and colony formation assays. HCT116 cells were treated with MSSV for 24 h. MSSV inhibited the growth of HCT116 cells in a dose-dependent manner (Figure 2A). The IC_50_ concentration was observed at 24.05 ± 1.07 μM. In addition, a cell counting assay was performed to determine the anti-proliferative activity on HCT116 cells. The cells were treated with various concentrations of MSSV for 24 h. The relative cell numbers reduced by 16, 27, 35, 47, and 53% at concentrations of 10, 12.5, 15, 17.5, and 20 μM of MSSV (Figure 2B). Based on the results, the concentrations of MSSV were selected for further experiments to lower than approximately 24 μM. A colony formation assay was conducted to further investigate the inhibitory effect of MSSV against human CRC cells. MSSV also exhibited a dose-dependent inhibitory effect on colony formation of cells as shown in Figure 2C. The relative colony formation was suppressed by 37% and 77% at the concentrations of five and 7.5 μM, respectively. After treatment with MSSV for 12 days, MSSV completely inhibited the colony formation at 10 μM or higher concentrations. These results indicate that MSSV has high inhibitory potency on proliferative ability on HCT116 cells.

### 3.2 MSSV induces cell cycle arrest at the G0/G1 phase on HCT116 cells

**FIGURE 3:**
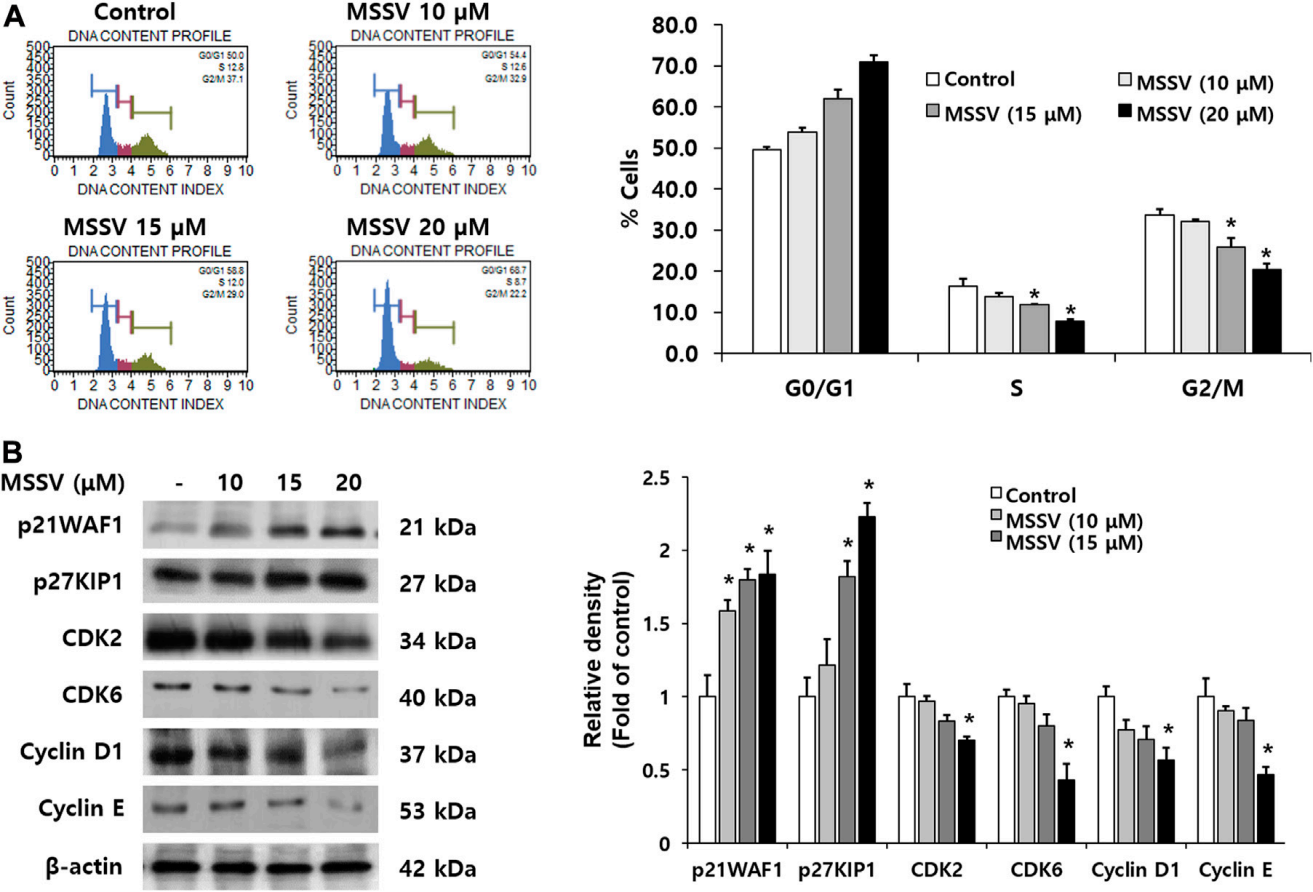
MSSV induces G0/G1 phase arrest in HCT116 cells **(A)** The effect of MSSV on cell cycle progression in HCT116 cells was estimated using the Muse^®^ Cell Analyzer. Cells were treated with MSSV at concentrations of 10, 15, and 20 μM. MSSV caused G0/G1 phase arrest in HCT116 cells. The bar represents the percentage of cells in each cell cycle phase. **(B)** The expression levels of proteins related to checkpoint control at G0/G1 phase in the presence or absence of MSSV were examined by immunoblotting. The expression level of proteins in cells was affected by the treatment with MSSV (0, 10, 15, and 20 μM). The bar represents the relative fold changes in proteins at different concentrations of MSSV in comparison with the control. Data are presented as mean ± SD and the * (asterisk) indicated statistically significant difference. A *p*-value of <0.05 was considered statistically significant.

To further examine the effect of MSSV on HCT116 cells, a cell cycle progression was measured using the Muse^®^ Cell Analyzer. MSSV treatment exhibited cell cycle arrest at the G0/G1 phase with a concomitant decrease in the number of cells in the S and G2/M phase (Figure 3A). MSSV increased the cell population in the G0/G1 phase by 49.6, 53.8, 62.0, and 71.0% at 0, 10, 15, and 20 μM, respectively. We subsequently investigated the expression level of cell cycle regulatory proteins involved in MSSV-induced G0/G1 cell cycle in HCT116 cells using immunoblotting. Cells were treated with different concentrations of MSSV for 24 h. The level of cyclin D1, cyclin E, CDK2, and CDK6 expression was downregulated in the presence of MSSV (Figure 3B). Moreover, the expression level of negative regulators, such as p21WAF1 and p27KIP1, was increased by treatment with MSSV (Figure 3B). These results demonstrated that MSSV inhibits cell cycle progression in the G0/G1 phase by modulating cell cycle regulatory proteins on HCT116 cells.

### 3.3 MSSV induces an intrinsic apoptosis pathway in HCT116 cells

**FIGURE 4:**
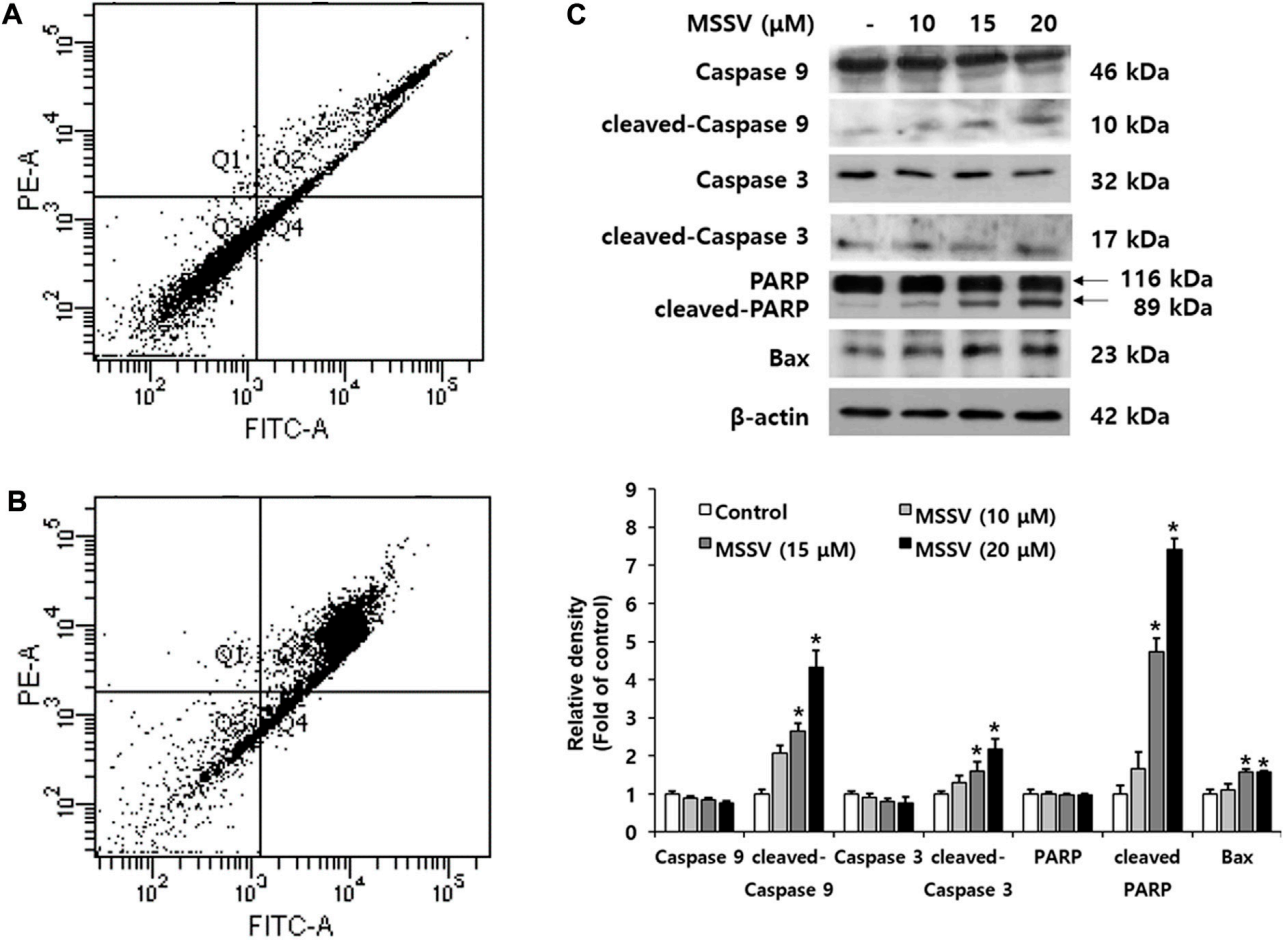
MSSV contributes a caspase-dependent intrinsic apoptotic pathway in HCT116 cells. **(A)** Control, and **(B)** HCT116 cells were treated with 20 μM MSSV. HCT116 cells were stained by both Annexin V and PI staining. **(C)** MSSV-induced apoptosis factors in HCT116 cells were determined by immunoblotting. The cells were treated with MSSV at concentrations of 10, 15, and 20 μM. The expression of pro-apoptotic proteins was increased by MSSV treatment in HCT116 cells. The bar represents the relative fold changes in proteins at different concentrations of MSSV in comparison with the control. Data are presented as mean ± SD and the * (asterisk) indicated statistically significant difference. A *p*-value of <0.05 was considered statistically significant.

Apoptosis is a crucial biological adjustment for maintaining homeostasis (Ha et al., 2017). To examine whether MSSV leads to apoptosis in HCT116 cells, cell flow cytometry was applied to Annexin V and PI staining. MSSV-treated cells had a higher number of stained cells than untreated cells. The accumulated cells in Q2 (late apoptosis) were remarkably increased and the percentage of cells in Q3 (viable cell) were decreased in HCT116 MSSV-treated cells (Figures 4A,B). These results suggest that MSSV induced apoptosis in HCT116 cells. To understand the apoptosis regulation by MSSV, the expression of apoptotic pathway molecules was examined using immublotting (Figure 4C). MSSV treatment exhibited an effective induction in the cleavage of caspase-3, caspase-9, and PARP with diminished pro-form of caspase-3 and caspase-9. It is well-known that PARP-1 [poly (ADP-ribose)polymerase-1] is essential for apoptotic signaling. A distinct event in apoptosis is a proteolytic cleavage of PARP-1, which is a nuclear enzyme involved in DNA repair, DNA stability, and transcriptional regulation. Caspase-3 cleaves the PARP-1 (116 kDa) to 85 and 24 kDa fragment (Los et al., 2002). Activation of caspases-3 and caspase-9 was observed in HCT116 cells treated with MSSV. Both caspase-3 and caspase-9 were included in the intrinsic apoptosis pathway (Zhang et al., 2016). Moreover, the expression of Bax, a pro-apoptotic protein, was significantly increased in cells treated with MSSV. Therefore, these results indicate that MSSV might stimulate apoptosis *via* the caspase-dependent intrinsic apoptosis pathway in HCT116 cells.

#### 3.4 MSSV induces G0/G1 phase arrest and apoptosis *via* suppression of the AKT signaling pathway

**FIGURE 5:**
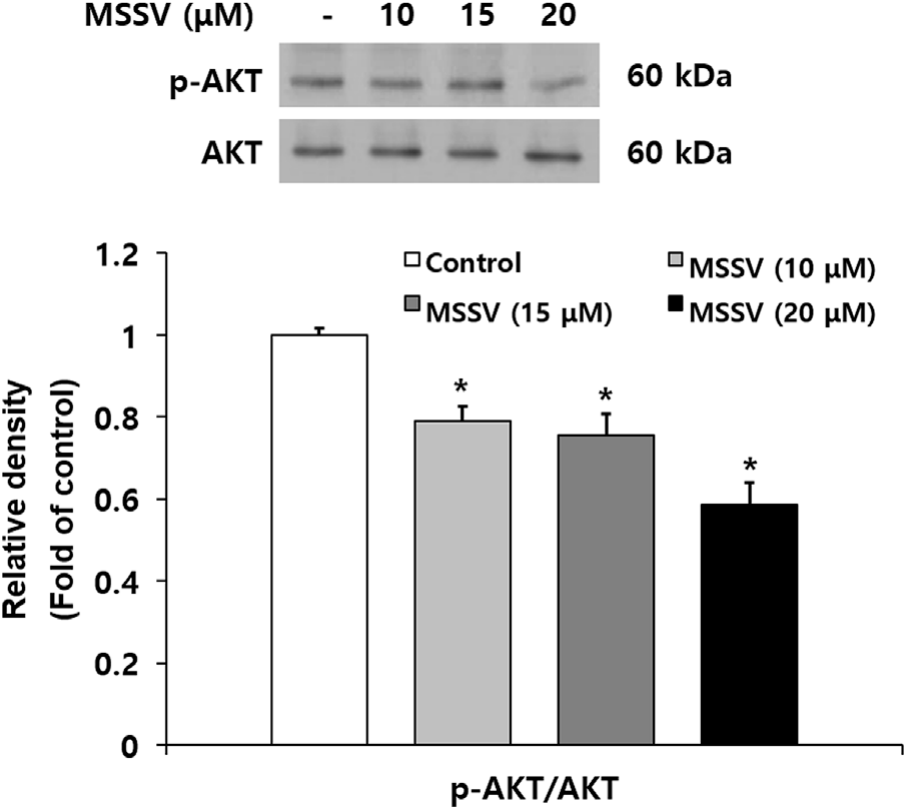
MSSV inhibited the phosphorylation of AKT in HCT116 cells. Cells were treated with MSSV at concentrations of 10, 15, and 20 μM for 24 h. The phosphorylation of AKT in cells treated with MSSV revealed a statistically significant reduction in comparison to the control group. The bar represents the relative fold changes in proteins at different concentrations of MSSV in comparison with the control group. Data are presented as mean ± SD and the * (asterisk) indicates a statistically significant difference. A *p*-value of <0.05 was considered statistically significant.

Many signaling pathways are involved in regulation for cell survival (Zhang et al., 2016). The AKT signaling pathway plays an important role in human cancer progression and development, and participates in reducing cell proliferation and promoting cell death (Bauer et al., 2015; Gao et al., 2020). To examine the effect of MSSV on AKT signaling, the level of AKT phosphorylation (p-AKT) was estimated by immunoblotting. The expression level of p-AKT (Ser473) was reduced in the treatment with MSSV and p-AKT/AKT ratio was expressed in Figure 5. AKT triggers a signaling network that positively regulates cell cycle transition *via* activation of series of cyclins and cyclin dependent kinases (CDKs) (Liang and Slingerland, 2003). The decrease of p-AKT stimulates G0/G1 phase arrest in HCT116 cells by downregulating CDK 2, 6, and cyclin D and E. Moreover, it is known that the reduction of p-AKT contributes to caspase-mediated apoptosis. Caspase-3 is a downstream protein of the AKT pathway (Schlaepfer and Hunter, 1996; Gan et al., 2006; Zhao et al., 2011; Tian et al., 2015). In the present study, MSSV induced the apoptosis of HCT116 cells by stimulating the activation of caspase-3, cascase-9, PARP, and Bax. These results imply that MSSV inhibits the phosphorylation of AKT, which may lead to the G0/G1 phase arrest and apoptosis in human CRC HCT116 cells.

MSSV inhibits the migration and invasion ability of HCT116 cells by impeding transcription factor-mediated MMP-9 expression.

**FIGURE 6:**
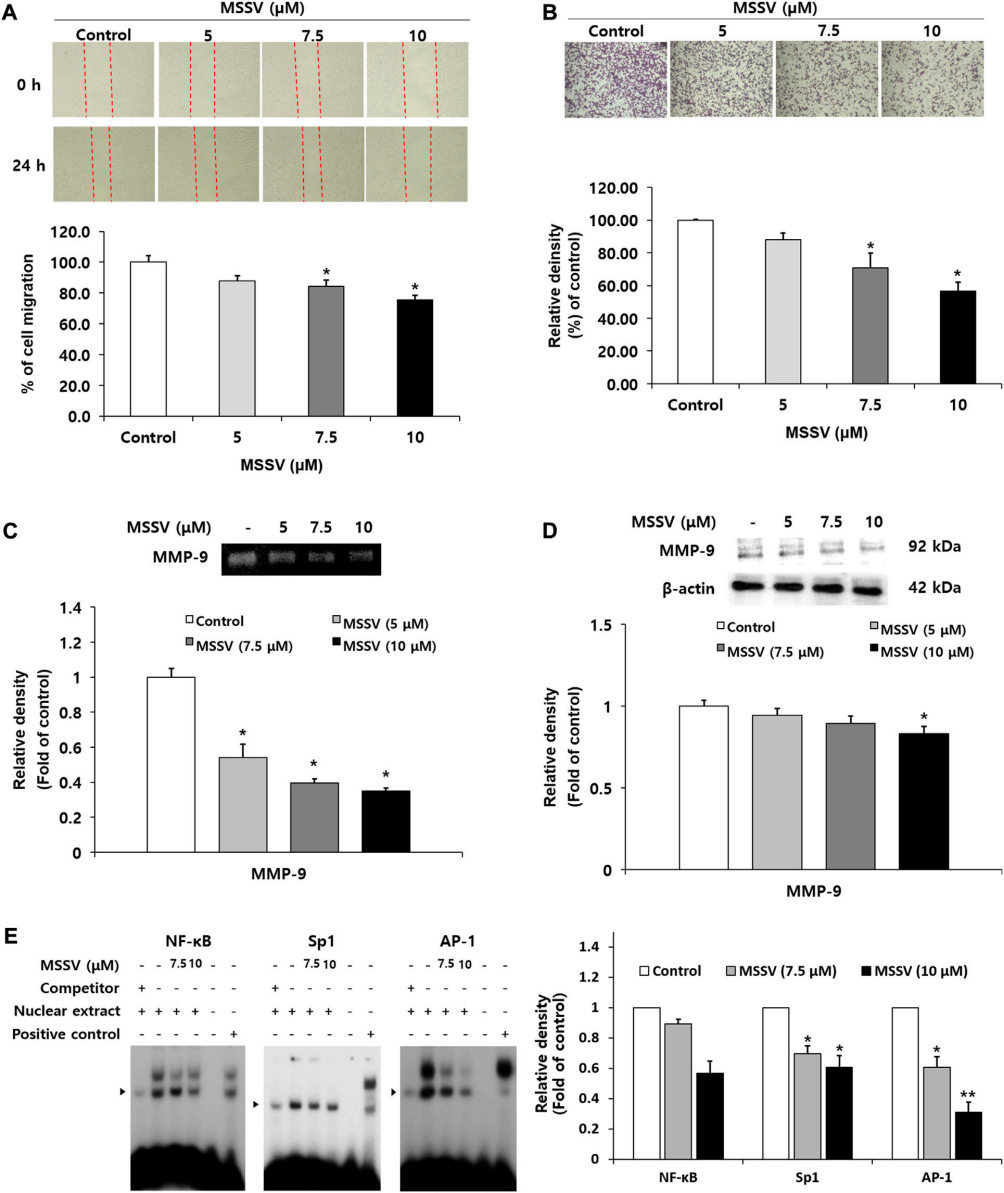
MSSV suppressed migration and invasion ability of HCT116 cells through decreased transcription factor-mediated MMP-9 expression **(A)** The inhibitory effect of MSSV on cell migration was determined by a wound-healing assay. Cells were plated on a 6-well plate and then incubated with MSSV (0, 10, 12.5, 15, 17.5, and 20 μM) for 24 h. The wound closure (%) was calculated as a percentage of migration. **(B)** The invasive ability of cells was estimated using a transwell invasion assay. Cells were treated with various concentrations of MSSV (0, 5, 7.5, 10, 12.5, and 15 μM) for 24 h. The relative density of invading cells is represented by a bar graph. **(C)** Gelatin zymography analysis was performed to analyze the activity of MMP-9. The bar represents the proteolytic activity of MMP-9. **(D)** MSSV reduced the expression level of the MMP-9 protein in HCT116 cells. Cells were treated with MSSV at concentrations of 5, 7.5, and 10 μM. The relative density of MMP-9 is expressed by a bar. **(E)** The binding ability of transcription factors AP-1, Sp-1, and NF-κB was verified using an EMSA experiment. The unlabeled AP-1, Sp-1, and NF-κB oligonucleotides were used as a 50x competitor. The * (asterisk) indicates a statistically significant difference and a *p*-value of <0.05 was considered statistically significant.

A wound-healing assay was performed to examine the inhibitory effect of MSSV on cell migration. Cell migration ability was decreased by 12, 15.7, and 24.5% at the concentrations of 5, 7.5, and 10 μM MSSV in a dose-dependent manner (Figure 6A). To determine the invasive ability through a thin layer matrix, a transwell invasion assay was performed (Kramer et al., 2013). The invasive ability of cells was suppressed by MSSV treatment at concentrations of 5, 7.5, and 10 μM through a transwell invasion assay (Figure 6B). It has been recognized that the matrix metalloproteinases (MMPs) have tumor-promoting effects that are involved in cell invasion, cell migration, and cancer metastasis (Kessenbrock et al., 2010; Franceschelli et al., 2020). In the current study, MSSV treatment inhibited the activity of MMP-9 in a dose-dependent manner as shown by a gelatin zymography (Figure 6C). In addition, the expression level of MMP-9 protein was decreased in the cells treated with MSSV (Figure 6D). To elucidate the MMP-9 regulation in MSSV-treated cells, binding activity of main transcription factors AP-1, Sp-1, and NF-κB, which are located in the promoter region of the MMP-9 gene, was examined through an EMSA experiment. MSSV treatment abolished the transcriptional binding ability of AP-1, Sp-1, and NF-κB in HCT116 cells (Figure 6E). These results indicate that MSSV suppressed MMP-9 expression *via* decreased binding activity of transcription factors, which lead to repression of the migratory and invasive properties of HCT116 cells.

The effect of cyclin D1 expression and AKT phosphorylation on the MSSV metabolites reacted with rat liver S9 fractions.

**FIGURE 7:**
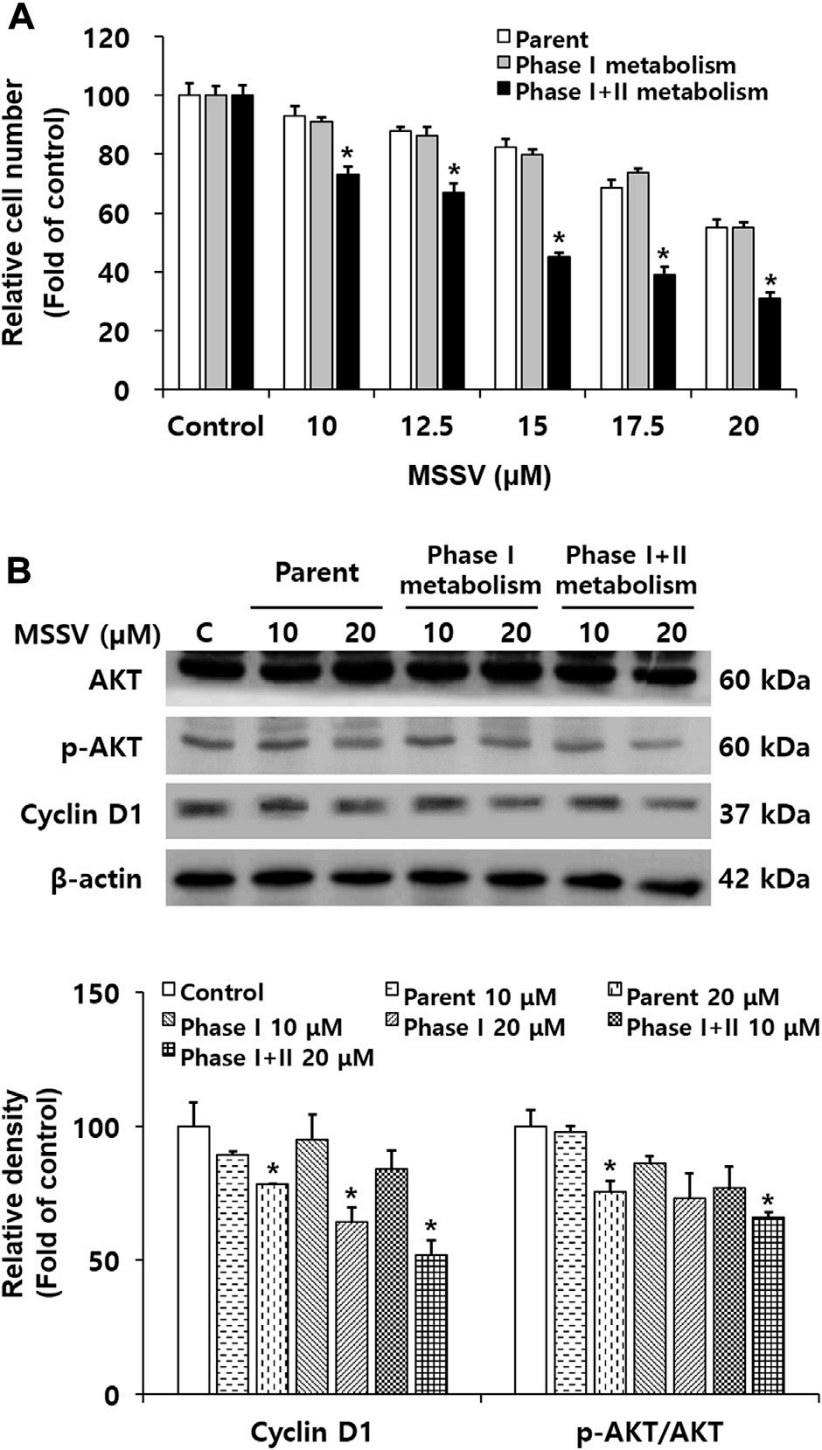
The anti-proliferative effects of MSSV were determined with treatment of S9 fractions against CRC cells **(A)** Cells were treated with various concentrations of MSSV and S9 fractions for 24 h. The trypan blue staining method was carried out for the cell counting assay. The bar represents the relative cell numbers at different concentrations of MSSV and statistically significant differences between the control and phase II metabolism group. **(B)** Western blotting was conducted to examine the sole and/or combination effect between MSSV and S9 fractions. Relative densities of cyclin D1 an p-AKT/AKT as a group are expressed as a bar graph. S9 fractions cause MSSV to more effectively inhibit the proliferation of HCT116 cells. Data are presented as mean ± SD and the * (asterisk) indicates a statistically significant difference. A *p*-value of <0.05 was considered statistically significant.

In the current study, rat pooled liver S9 fractions were used in the cell counting assay and immunoblotting to determine the *in vitro* metabolism of MSSV in HCT116 cells. The relative cell numbers were decreased in HCT116 cells treated with MSSV and MSSV metabolites. MSSV exhibited a dose-dependent growth inhibition effect in all treatment groups compared to the control group (Figure 7A). In addition, the MSSV metabolites showed the strongest inhibitory effect on the proliferation of HCT116 cells. The expression of cyclin D1 significantly decreased in the MSSV metabolite-treated group compared to the MSSV-treated group (Figure 7B). Similarly, levels of AKT phosphorylation were shown to be decreased by MSSV phase I + II metabolite (Figure 7B). These results suggest that the phase II metabolic process with S9 fractions enhances the anti-proliferative effect of MSSV on HCT116 cells.

### 3.5 MSSV suppressed the tumor growth of xenograft mice *in vivo*

**FIGURE 8:**
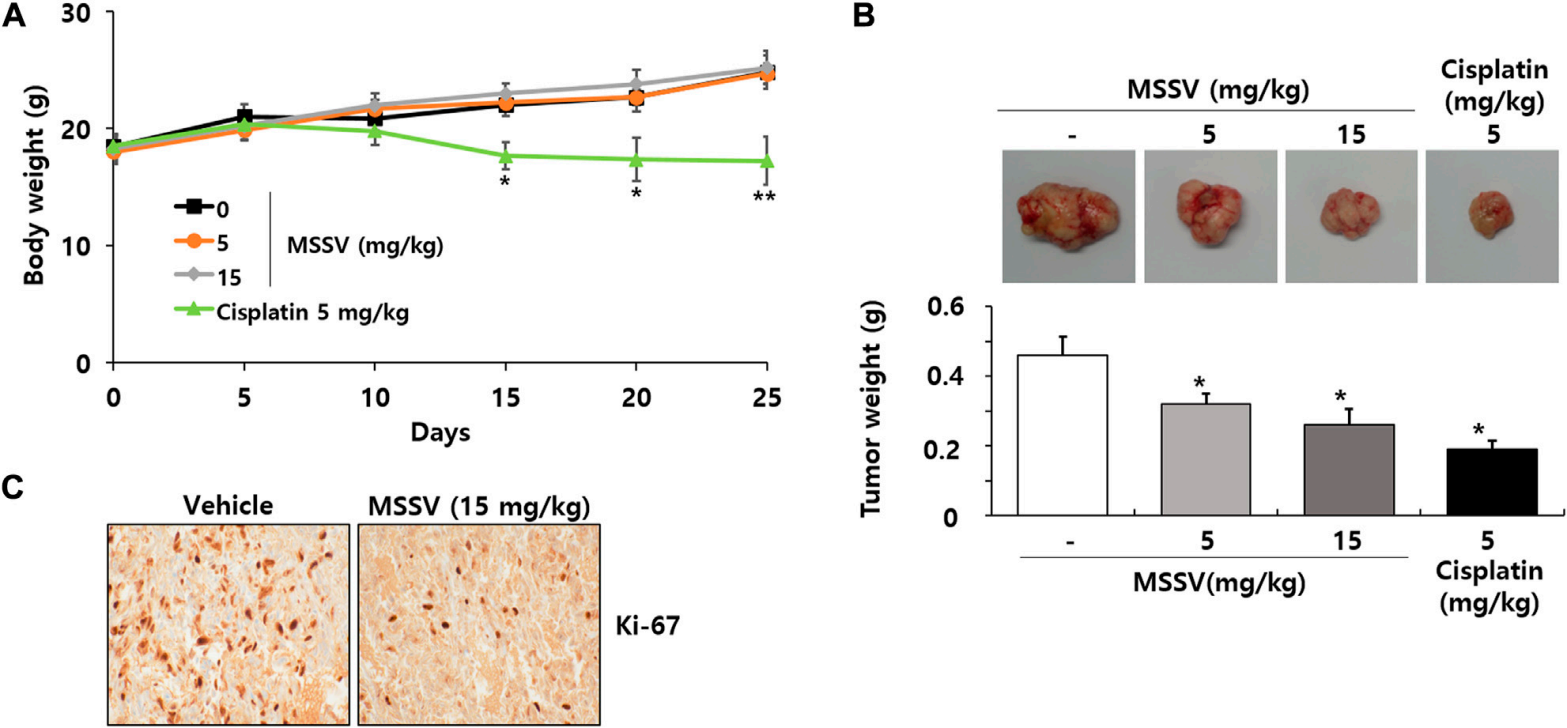
Oral injection of MSSV inhibited HCT116 cell growth in xenograft mice **(A)** Measurement of body weight change in mice after treatment with MSSV (0, 100, 200, and 400 mg/kg) and cisplatin (5 mg/kg). **(B)** The images and weights of tumors isolated from xenograft mice. **(C)** Ki-67 staining from tumor tissues. Data are presented as mean ± SD and the * (asterisk) indicates a statistically significant difference. A *p*-value of <0.05 was considered statistically significant.

Finally, an animal experiment was performed to demonstrate the *in vivo* antitumor effect of MSSV using xenograft mice bearing HCT116 cells. No significant body weight change was observed between control mice and MSSV-treated mice (Figure 8A). However, the body weight of cisplatin-treated mice was apparently decreased by 25% compared to control mice (Figure 8A), suggesting that cisplatin is obviously toxic to mice. In addition, oral administration of MSSV remarkably suppressed the tumor growth of xenograft mice compared with that of tumors from control mice (Figure 8B). Reduced proliferative capacity of tumor cells derived from MSSV-treated xenograft mice was validated by Ki-67 staining (Figure 8C). These results suggest that MSSV could exert an effective antitumor efficacy *in vivo* against CRC.

## 4 Discussion

MSSV has been shown to exhibit anticancer effects (Wesson and Hamann, 1996; Cueto et al., 2000; Simmons et al., 2009; Thundimadathil, 2012). Our previous study demonstrated that MSSV suppresses anti-tumor and anti-angiogenic activity against bladder cancer both *in vitro* and *in vivo* (Song et al., 2021). Other studies have suggested the cytotoxicity of MSSV against HCT116 cells without examining the mechanisms involved (Cueto et al., 2000). We herein report the mechanisms of action of MSSV in CRC. We found that MSSV induces a G0/G1 phase arrest, apoptosis, and AKT signaling inhibition in human CRC HCT116 cells. The abnormal cell cycle leads to uncontrolled cell proliferation as a characteristic of cancer progression (Deep and Agarwal, 2008; Ha et al., 2017). The AKT signaling pathway plays a significant role in human cancer progression. Inhibition of AKT signaling resulted in decreased cell proliferation and promoted cell death (Manning and Cantley, 2007; Bauer et al., 2015; Gao et al., 2020). AKT inhibition triggers a network that negatively regulates cell cycle transition *via* the inactivation of a series of cyclins and CDKs. It also stimulates caspase-mediated apoptosis in malignancies (Liang and Slingerland, 2003; Tripathi et al., 2017). In the present study, the level of p21 and p27 largely increased in HCT116 cells incubated with MSSV, which stimulates the reduction of CDK2, CDK6, cyclin D1, and cyclin E that induces the suppression of the G1 to S-phase progression. In addition, MSSV induced apoptosis in HCT116 cells by increasing the level of the cleaved form of caspase-3, caspase-9, and PARP. Caspase-3 is known as a downstream protein of the AKT pathway (Schlaepfer and Hunter, 1996; Gan et al., 2006; Zhao and Guan, 2009; Tian et al., 2015). The inhibition of cell cycle progression and induction of apoptosis by AKT pathway suppression was observed following treatment with cetylcoptisine, a new coptisine derivative, in human A549 cells (Han et al., 2019). Surfactin is a cyclic lipopeptide with anti-inflammatory, anti-viral, anti-mycoplasma, anti-fibrinogenic, and anti-hypercholesterolemic properties (Kim et al., 2007). This cyclic lipopeptide upregulates the level of p21, cleaved caspase 3, and cleaved PARP and downregulates the phosphorylation of AKT and the expression of CDK2 and cyclin E in CRC cells. Our results indicate that MSSV exhibits a similar regulatory mechanism to surfactin in the inhibition of the proliferation of human CRC cells.

Cell migration and invasion are necessary for many biological processes and the development of many diseases (Yamaguchi and Condeelis, 2007). The invasive and migratory process begins with a secretion of MMP to degrade the basement membrane. The movement of cancer cells by MMP action into tissue surrounding the vasculature and tumor is the essential step in cancer metastasis (Sahai, 2005). A recombinant analgesic-antitumor peptide (rAGAP), a polypeptide, led to the inhibition of migration *via* MMP-9 inactivation in SHG44 cells (human malignant glioma cells) (Zhao et al., 2011). Relaxin is a short circulating peptide hormone, which decreases the *in vitro* invasive ability in osteosarcoma MG-63 cells by reducing the level of MMP-9 (Ma et al., 2013). Similarly, MSSV exhibited cell migration and invasion suppression using a wound-healing migration and transwell invasion assay in HCT116 cells. In addition, active MMP-9 was inhibited by treatment of MSSV with cells. MSSV treatment suppressed the expression level of MMP-9 protein in HCT116 cells through an immunoblotting. Furthermore, MSSV suppressed the binding activity of transcription factors AP-1, Sp-1, and NF-κB motifs, which are main elements exist in the promoter region of the MMP-9 gene. These results demonstrate that MSSV impeded the metastatic ability of HCT116 cells *via* MMP-9 inhibition mediated by reduced the binding activity of transcription factors.

The toxicity of the parent compounds can be determined only if metabolic enzymes to convert the parent compound to metabolites are present in an *in vitro* system (Chitrangi et al., 2017; Shao et al., 2020). The liver is considered a major site of metabolism because metabolism centered on the liver has been studied, including primary cultures of hepatocytes, subcellular fractions, precision-cut liver tissue slices and drug-metabolizing enzymes (Rodrigues, 1994). The *in vitro* models most often used are subcellular fractions (cytosols, microsomes, or S9 fractions), subcellular organelles (liver slices and hepatocytes), and metabolizing recombinant enzymes (CYP and UDP-glucuronosyltransferases) (Jia and Liu, 2007). The liver S9 fraction includes both cytosolic and microsomal fractions. The S9 fractions contains both phase I and phase II metabolic activity comparing cytosol and microsomes, which indicate that metabolic profiles are expressed better (Brandon et al., 2003). The liver S9 fraction provides the same quality of hepatocyte and is more efficient and cost effective. We examined the effect of MSSV metabolites on the proliferation of HCT116 cells using a cell counting assay with the S9 fraction (Lassila et al., 2015). Interestingly, MSSV metabolites formed in phase I and phase I + II metabolism exhibited stronger inhibitory ability on the proliferation of cell. The expression level of cyclin D1 was more decreased by MSSV metabolites than by MSSV. Similar tendency was observed for the expression level of AKT phosphorylation. It was confirmed that the phase I + II metabolic process enhances the inhibitory activity of MSSV on the proliferation of HCT116 cells. These results suggest that MSSV may inhibit the progression of colorectal cancer *in vivo*. In general, the liver plays a crucial role in protecting organisms from toxic chemicals through conversion from lipophilic molecules to more water-soluble metabolites, which can be efficiently removed from the body *via* urination. This protective ability of the liver is derived from the expression of a variety of xenobiotic biotransforming enzymes with fundamental features including catalyzing the oxidation, reduction, and hydrolysis (Phase I metabolism) and/or conjugation of functional groups on chemical molecules and drug (Phase II metabolism). Some chemicals can be converted to toxic metabolites by certain enzymatic reactions (Grant, 1991). Cases for several compounds were reported that activated their activity or toxicity by phase II metabolism (Yoshihara et al., 2001; Cui et al., 2005; Gramec et al., 2014; Yang et al., 2019; Geburek et al., 2020). Thiophene is a sulfur-containing heteroaromatic ring commonly used as a building block in drugs, which can lead to forming the reactive metabolites. As a result, thiophene metabolism causes the toxicity of drugs containing the thiophene moiety. The addition of the thiophene did not affect on rat cerebellar granule cell cultures, but the addition of thiophene with the S9 fraction and corresponding cofactors (NADPH and glucose-6-phosphate) extensively reduced the viability of that cell (Gramec et al., 2014). Similarly, it was confirmed that the metabolic process with liver S9 fractions enhances the inhibitory activity of MSSV on the proliferation of HCT116 cells.

Finally, we assessed the antitumor efficacy of MSSV *in vivo* using an HCT116 xenograft mouse. Oral administration of MSSV suppressed the tumor growth of xenograft mice. Our previous study revealed that MSSV did not show any adverse toxic signs in an acute toxicity test (Song et al., 2021). When all the results were combined in current study, MSSV is suggested to possess an inhibitory activity on the progression of CRC.

## 5 Conclusion

We demonstrated the antitumor efficacy of MSSV and its mechanism of action in CRC. MSSV significantly inhibited the proliferation of CRC HCT116 cells. Notably, MSSV induced G0/G1 cell cycle arrest *via* downregulation of CDKs and cyclins by upregulating p21WAF1 and p27KIP1. In addition, MSSV treatment triggered apoptosis through stimulation of cleaved caspase-3, cleaved PARP, cleaved caspase-9, and pro-apoptotic Bax. A reduced level of AKT phosphorylation was observed in the presence of MSSV. MSSV decreased the metastatic potential of HCT116 cells by regulating transcription factor-associated MMP-9 expression. The *in vitro* metabolites with rat liver S9 fractions enhanced the anti-proliferative potential of MSSV *via* reduction of both cyclin D1 expression and AKT phosphorylation. Furthermore, MSSV suppressed tumor growth of xenograft mouse. Taken together, our study provides insights for the development of MSSV as an antitumor agent against CRC.

## Data Availability

The original contributions presented in the study are included in the article/supplementary material, further inquiries can be directed to the corresponding authors.
